# Global, regional, and national epidemiology of otitis media in children from 1990 to 2021

**DOI:** 10.3389/fped.2025.1513629

**Published:** 2025-07-02

**Authors:** HanYu Wang, XiaoYu Zeng, Xin Miao, BoWen Yang, ShiPeng Zhang, QinWei Fu, QinXiu Zhang, Mi Tang

**Affiliations:** ^1^ Clinical Medical College, Chengdu University of Traditional Chinese Medicine, Chengdu, Sichuan, China; ^2^ School of Integrated Traditional Chinese and Western Medicine, Southwest Medical University, Luzhou, Sichuan, China; ^3^ Oncology Department, Dongguan Hospital, Guangzhou University of Chinese Medicine, Dongguan, Guangdong, China; ^4^ Department of Health Research Methods, Evidence and Impact (HEI), McMaster University, Hamilton, ON, Canada; ^5^ Otolaryngology Department, Hospital of Chengdu University of Traditional Chinese Medicine, Chengdu, China

**Keywords:** otitis media, global burden of disease, disability-adjusted life years, sociodemographic index, risk factors

## Abstract

**Background:**

Otitis media(OM) is a prevalent ear condition, particularly among children, with a significant impact on quality of life. This study aimed to elucidate the global prevalence and disability-adjusted life years (DALYs) associated with OM in the 0–14 age group from 1990–2021, using data from the 2021 Global Burden of Disease (GBD) study.

**Methods:**

Our study conducted a comprehensive analysis of OM data from the GBD 2021 report, examining the prevalence and DALYs related to OM across 204 countries and territories over a 32-year period. Data were stratified by age, sex, year, geographic region, and sociodemographic index (SDI). Temporal trends were evaluated using estimated annual percent change (EAPC) calculations. Additionally, a global risk attribution analysis for childhood OM was conducted, and a Bayesian age-period-cohort (BAPC) model was applied to project the global burden of childhood OM from 2021–2035.

**Results:**

In 2021, the global burden of OM in children remained significant, with an incidence of 297,243,470 cases and an age-standardized prevalence of 14,775 cases per 100,000 population. OM resulted in 1,035,749 DALYs globally, with an age-standardized DALY rate of 51.48 per 100,000 population. Regionally, the highest age-standardized prevalence of OM was observed in Eastern Sub-Saharan Africa, South Asia, and Western Sub-Saharan Africa, while Central Europe, East Asia, and High-income Asia Pacific exhibited the lowest prevalence. Key risk factors identified include secondhand smoke, particulate matter pollution, low birth weight, and short gestation. Additionally, a significant inverse association was found between the SDI and the burden of OM, with age-standardized DALY rates decreasing markedly as SDI increased.

**Conclusion:**

OM in children represents an escalating global health challenge, marked by a rising incidence. Although there has been a global decline in DALYs, the burden of DALYs associated with OM in children remains significant, particularly in regions with a low SDI. A more comprehensive understanding of the epidemiology of OM in children could enhance prevention and control efforts.

## Introduction

Otitis media (OM) is a persistent condition characterized by inflammation of the middle ear and the presence of middle ear effusion (MEE). It is typically classified into acute OM (AOM) and chronic OM (COM) (1, 2). OM is one of the most prevalent pediatric diseases globally (3). A recent systematic review on the global burden of OM estimated the average incidence of AOM at 10.8 new episodes per 100 people per year (4), while the incidence of chronic OM is estimated at 4.8 new episodes per 1,000 people (all ages) per year (4). OM is the leading cause of hearing loss in children (5–7), with hearing loss potentially being temporary or permanent and often persisting into adulthood (8). However, disparities in the treatment of children with OM persist (9). Previous studies have documented differences in diagnosis, treatment, and complications, with children from lower socioeconomic backgrounds exhibiting a higher prevalence of OM and a greater likelihood of complications (10, 11). Understanding the global and regional burden of OM in children is crucial for the equitable allocation of healthcare resources, particularly in low- and middle-income countries.

The Global Burden of Disease (GBD) study offers comprehensive epidemiological data and valuable insights for public health decision-making through systematic analysis of a wide range of diseases and risk factors (12). The GBD database includes extensive data from 1990–2021, encompassing incidence and disability-adjusted life years (DALYs), providing a valuable resource for assessing global trends and determining influencing factors of OM in children.

According to the GBD study, the number of children with OM increased by 40,940,535 between 1990 and 2021. This highlights OM in children as a critical target for reducing the burden of non-communicable diseases in this population. A recent study by Huang et al. (13) utilized GBD 2021 data to assess the global burden of OM across all age groups from 1992–2021 and forecast trends through 2036. While their findings provided valuable macro-level insights, particularly regarding the overall burden and temporal changes across regions and SDI levels, their analysis did not focus specifically on the pediatric population nor did it include in-depth exploration of specific pediatric risk factors. In contrast, our study narrows the focus to children aged 0–14 years and incorporates comprehensive assessment of key modifiable risk factors. We use data from GBD 2021 to present a comprehensive evaluation of the global, regional, and national burden of OM in children, along with its associated risk factors. This analysis aims to inform healthcare professionals and support the development of new preventive and therapeutic strategies to reduce the health burden of OM in children.

## Methods

### Data acquisition and sources

The data for this study were obtained from the GBD 2021 dataset, which provides comprehensive information on the global and regional burden of 369 diseases, injuries, and 88 risk factors across 204 countries and territories from 1990–2021 (14). Specifically, we extracted incidence numbers and DALYs for OM among children aged 0–14 years. DALY is a comprehensive measure of disease burden, combining years of life lost due to premature death and years lived with disability. To summarize the age distribution of the burden of OM, patients were categorized into five age groups: younger than 1 year, 12–23 months, 2–4 years, 5–9 years, and 10–14 years. Additionally, sociodemographic index (SDI) data were utilized to assess the impact of socioeconomic factors on disease burden. The SDI is a composite indicator that represents the geometric mean of three factors: per capita income (with a lagged distribution), average years of schooling, and the fertility rate of women under 25 in a given area. The Global Development Report 2021 classifies 204 countries and territories into five groups based on SDI quintiles: low SDI, low-middle SDI, middle SDI, middle-high SDI, and high SDI regions. All data are available for download via the Global Health Data Exchange (GHDx) platform (http://ghdx.healthdata.org/gbd-results-tool).

### Risk factor analysis

In this study, we used data from the GBD 2021 study to analyze the risk factors contributing to OM burden and performed a comprehensive analysis to assess attributable DALYs for each risk factor.

### BAPC model for forecasting

To predict the future burden of OM, we employ a BAPC model. The model takes into account the effects of age, period, and cohort, providing a comprehensive approach to understanding future trends in disease burden. Previous studies have shown that BAPC has higher coverage and accuracy compared to other forecasting methods (15).

### Statistical analysis

Age-standardized rate (ASR) and EAPC were used to study trends in incidence and DALYs of OM to eliminate heterogeneity. Based on a previous study, we calculated the EAPC using linear regression (16). When the upper limit of the 95% confidence interval is below 0, it indicates a downward trend, while the lower limit exceeds 0, it indicates an upward trend. If 95% CI contains 0, there is no statistically significant change in the trend patterns. In this study, the R software package (version 4.3.1) and JD_GBDR (V2.26, Jingding Medical Technology Co., Ltd.) was used for the drawing of the figures.

For GBD studies, the Institutional Review Board of the University of Washington reviewed and approved a waiver of informed consent (https://ghdx.healthdata.org/gbd-2021).

## Results

### OM in children: global trends

#### Global incidence patterns

**Figure 1:**
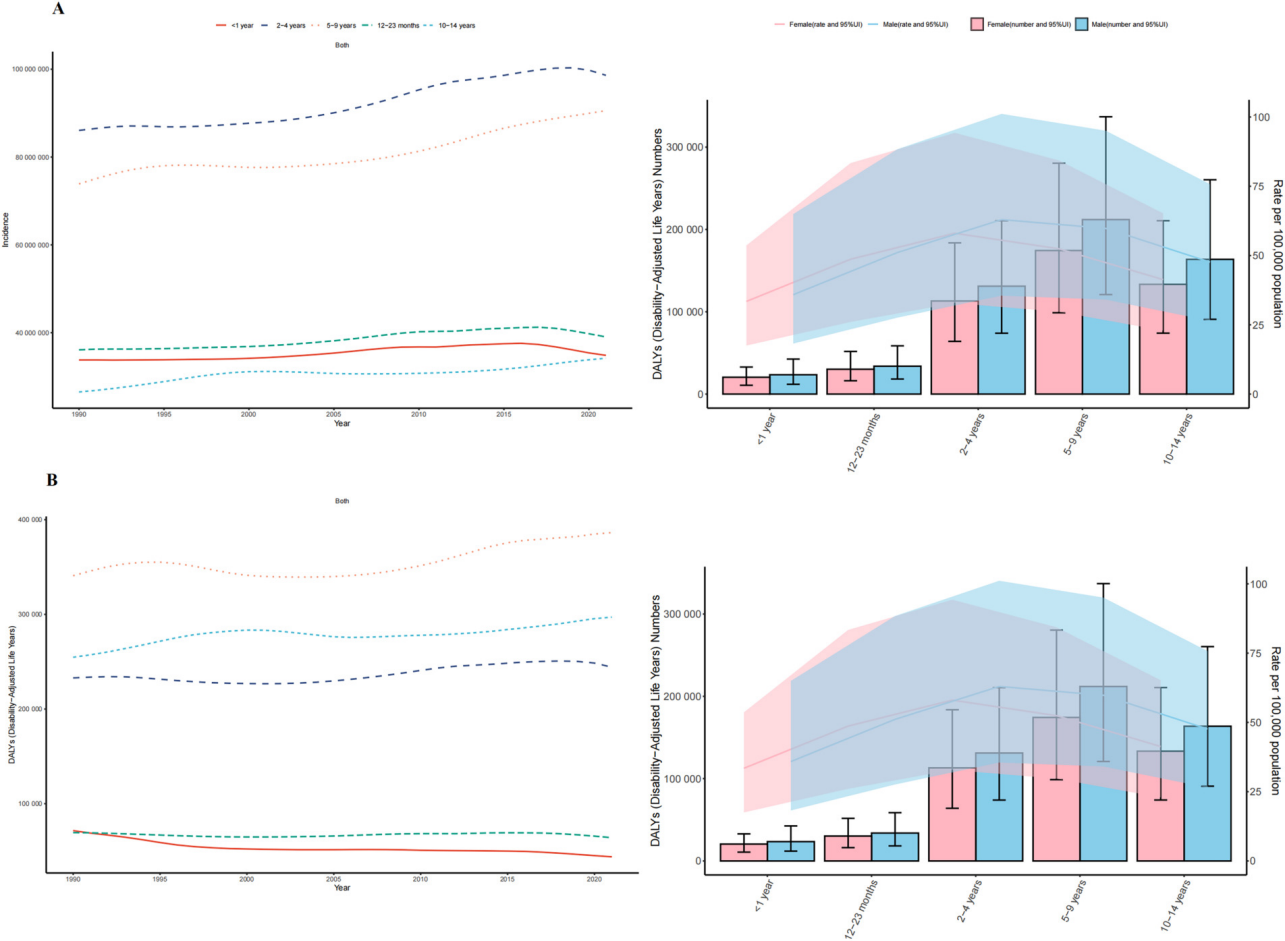
Trends in otitis media incidence and disability-adjusted life-years (DALYs) among children from 1990 to 2021. **(A)** Trends in incidence cases and incidence rate. **(B)** Trends in DALYs cases and DALYs rate.

A total of 51,558,104 children [27,747,118 male [53.82%]; 23,810,986 female [46.18%]] were included in the analysis. In 2021, the global incident cases of OM in children were 297,243,470 [95% uncertainty interval (UI), 205,198,444.24–431,726,180.40]. From 1990–2021, the global incident cases of childhood OM increased by 15.97% (95% UI, 13.99%–17.86%). Similarly, the OM-associated incidence rate increased from 14,737 per 100,000 in 1990–14,775 per 100,000 in 2021; the EAPC was 0.12 (95% CI, 0.08–0.17). From 1990–2021, the incidence of OM increased in children of all ages. The largest increase (29.02%) occurred in children aged 10–14 years. The smallest increase (3.11%) occurred among children aged <1 years. The incidence of OM was highest among children aged 2–4 years in both 1990 and 2021 (33.58% and 33.18%, respectively). In 2021, the incidence of OM was generally higher in girls than in boys. The male to female ratio of OM incidence among children of different ages exhibited a bimodal distribution, with a peak among children aged 12–23 months (Table 1 and Figure 1A).

**Table 1:** Incidence of otitis media in children between 1990 and 2021 at the global and regional level.

Location	Rate per 100,000 (95% UI)					
	1990		2021		1990–2021	
	Incident cases	Incidence rate	Incident cases	Incidence rate	Cases change	EAPC
Global	25,63,02934.67 (17,78,25908.10 to 36,89,74180.91)	14737.24 (10224.87 to 21215.76)	29,72,43469.52 (20,51,98444.24 to 43,17,26180.40)	14,774.55 (10,199.43 to 21,459.04)	0.16 (0.14 to 0.18)	0.12 (0.08 to 0.17)
High SDI	21,98,9645.21 (16,38,5874.97 to 30,07,4283.14)	11834.62 (8818.72 to 16185.70)	20,63,9456.11 (15,46,8152.07 to 27,90,5038.69)	11,962.37 (8,965.15 to 16,173.41)	−0.06 (−0.08 to −0.03)	0.10 (0.07 to 0.13)
High-middle SDI	32,26,5944.27 (22,18,3930.92 to 46,87,3547.34)	11792.11 (8107.48 to 17130.70)	26,47,9525.79 (17,97,8997.77 to 38,75,2392.92)	11,468.44 (7,786.81 to 16,783.89)	−0.18 (−0.20 to −0.16)	0.20 (0.09 to 0.32)
Middle SDI	77,82,9616.54 (53,57,9831.23 to 11,32,17937.25)	13483.67 (9282.49 to 19614.55)	75,39,6823.99 (51,48,2658.48 to 11,13,23365.56)	13,300.76 (9,082.06 to 19,638.57)	−0.03 (−0.06 to −0.01)	0.13 (0.07 to 0.20)
Low-middle SDI	82,01,3836.35 (56,16,6569.39 to 11,92,73506.41)	17371.69 (11896.87 to 25263.81)	94,82,1611.11 (65,15,6517.66 to 13,81,88242.00)	16,353.03 (11,236.96 to 23,832.08)	0.16 (0.13 to 0.18)	−0.15 (−0.16 to −0.13)
Low SDI	42,01,6185.10 (29,00,3873.93 to 60,09,2888.76)	18354.62 (12670.24 to 26251.36)	79,70,6870.98 (54,87,6647.51 to 11,46,78212.66)	17,319.09 (11,923.86 to 24,917.83)	0.90 (0.86 to 0.94)	−0.18 (−0.21 to −0.16)
Andean Latin America	21,22,759.92 (14,55,275.29 to 30,90,819.27)	14292.72 (9798.49 to 20810.75)	25,29,032.81 (17,28,564.20 to 37,01,655.09)	13,976.58 (9,552.83 to 20,457.02)	0.19 (0.18 to 0.20)	−0.06 (−0.10 to −0.02)
Australasia	622283.07 (431344.29 to 877045.40)	13569.31 (9405.76 to 19124.58)	755243.81 (520652.34 to 10,69,420.28)	13,177.88 (9,084.61 to 18,659.80)	0.21 (0.20 to 0.23)	−0.00 (−0.06 to 0.05)
Caribbean	16,38,475.86 (11,27,943.79 to 23,46,466.50)	14356.95 (9883.47 to 20560.63)	15,96,764.70 (11,02,733.82 to 22,92,449.83)	13,878.76 (9,584.74 to 19,925.51)	−0.03 (−0.04 to −0.01)	−0.09 (−0.13 to −0.04)
Central Asia	29,09,637.56 (19,65,028.57 to 42,92,380.77)	11642.64 (7862.88 to 17175.56)	31,78,153.40 (21,57,611.61 to 46,90,383.22)	11,483.51 (7,796.02 to 16,947.60)	0.09 (0.08 to 0.10)	0.10 (−0.03 to 0.23)
Central Europe	29,43,967.53 (20,06,594.41 to 42,85,111.59)	9985.10 (6805.79 to 14533.87)	17,39,627.60 (12,27,141.35 to 24,34,863.23)	9,827.73 (6,932.53 to 13,755.35)	−0.41 (−0.44 to −0.37)	0.15 (0.06 to 0.23)
Central Latin America	96,14,714.66 (66,27,024.02 to 13,77,4746.19)	14934.04 (10293.41 to 21395.60)	89,59,924.89 (61,25,843.63 to 13,01,1985.33)	14,113.46 (9,649.28 to 20,496.17)	−0.07 (−0.09 to −0.04)	−0.16 (−0.18 to −0.13)
Central Sub-Saharan Africa	44,10,771.99 (30,75,439.81 to 63,05,682.08)	17434.83 (12156.55 to 24925.00)	96,19,143.53 (66,53,328.74 to 13,88,6873.94)	16,392.09 (11,338.01 to 23,664.77)	1.18 (1.12 to 1.25)	−0.14 (−0.18 to −0.11)
East Asia	34,90,1969.67 (24,49,9879.50 to 51,22,2167.07)	10581.68 (7427.95 to 15529.69)	26,67,8404.84 (18,21,2689.22 to 39,94,0479.98)	9,978.75 (6,812.25 to 14,939.28)	−0.24 (−0.27 to −0.20)	0.13 (−0.04 to 0.29)
Eastern Europe	61,82,614.27 (42,15,097.08 to 91,70,699.44)	12013.95 (8190.70 to 17820.34)	40,95,570.68 (28,00,129.00 to 60,82,089.48)	11,555.03 (7,900.14 to 17,159.70)	−0.37 (−0.36 to −0.32)	0.29 (0.12 to 0.47)
Eastern Sub-Saharan Africa	17,02,1068.41 (11,59,6992.11 to 24,57,3647.69)	18793.12 (12804.35 to 27132.00)	31,90,4752.66 (21,66,1692.70 to 46,00,8249.13)	17,880.67 (12,140.06 to 25,784.82)	0.87 (0.83 to 0.92)	−0.16 (−0.19 to −0.14)
High-income Asia Pacific	32,70,219.38 (22,80,891.82 to 46,42,389.02)	9290.52 (6479.89 to 13188.78)	22,48,633.28 (15,44,431.53 to 32,58,413.23)	10,027.11 (6,886.93 to 14,529.92)	−0.31 (−0.34 to −0.15)	0.22 (0.16 to 0.28)
High-income North America	69,86,120.80 (56,45,959.08 to 86,49,053.86)	11326.83 (9153.98 to 14022.99)	73,06,111.61 (60,29,520.13 to 88,38,183.06)	1,1134.15 (9,188.69 to 13,468.95)	0.05 (0.01 to 0.09)	−0.08 (−0.11 to −0.04)
North Africa and Middle East	20,65,7741.82 (14,11,9634.52 to 30,06,7951.39)	14704.55 (10050.61 to 21402.90)	25,69,1621.12 (17,54,4252.77 to 37,52,7523.67)	14,014.48 (9,570.18 to 20,470.82)	0.24 (0.22 to 0.26)	0.02 (−0.03 to 0.08)
Oceania	345727.94 (236138.18 to 505872.64)	12900.95 (8811.57 to 18876.80)	659744.10 (450074.95 to 967314.36)	12,984.87 (8,858.23 to 19,038.37)	0.91 (0.90 to 0.91)	0.04 (0.01 to 0.06)
South Asia	82,08,5225.80 (55,95,2713.88 to 12,03,28509.68)	18941.50 (12911.31 to 27766.29)	89,04,4430.25 (60,92,6931.49 to 13,04,05120.07)	17,562.26 (12,016.64 to 25,719.84)	0.08 (0.06 to 0.12)	−0.21 (−0.22 to −0.20)
Southeast Asia	21,05,4175.68 (14,61,5367.87 to 30,44,6068.65)	12330.59 (8559.64 to 17831.04)	21,01,3794.01 (14,23,6535.42 to 30,97,6089.04)	12,171.07 (8,245.72 to 17,941.18)	0.00 (−0.03 to 0.03)	−0.00 (−0.02 to 0.01)
Southern Latin America	20,19,009.32 (13,89,642.10 to 28,52,289.60)	13526.40 (9309.94 to 19108.98)	18,17,915.72 (12,47,126.79 to 26,05,422.69)	12,541.21 (8,603.52 to 17,973.97)	−0.10 (−0.13 to −0.17)	−0.09 (−0.15 to −0.03)
Southern Sub-Saharan Africa	35,70,148.04 (24,36,125.86 to 51,84,060.83)	17255.99 (11774.80 to 25056.69)	40,01,874.46 (27,22,026.70 to 58,44,302.74)	16,628.85 (11,310.74 to 24,284.63)	0.12 (0.10 to 0.14)	0.01 (−0.04 to 0.06)
Tropical Latin America	79,29,819.63 (54,28,153.04 to 11,53,6341.88)	14790.58 (10124.51 to 21517.41)	76,22,359.31 (52,59,833.03 to 11,05,9050.80)	15,186.09 (10,479.21 to 22,033.03)	−0.04 (−0.05 to −0.02)	0.14 (0.10 to 0.17)
Western Europe	10,21,0222.99 (71,88,857.17 to 14,66,8013.73)	14376.89 (10122.54 to 20653.86)	96,62,354.18 (67,90,973.98 to 13,93,7495.57)	14,184.66 (9,969.38 to 20,460.71)	−0.05 (−0.06 to −0.05)	0.05 (0.01 to 0.09)
Western Sub-Saharan Africa	15,80,6260.34 (10,92,1263.50 to 22,57,9937.40)	17986.22 (12427.50 to 25694.11)	37,11,8012.58 (25,50,1999.36 to 53,28,6901.25)	17,283.18 (11,874.44 to 24,811.87)	1.35 (1.30 to 1.40)	−0.10 (−0.13 to −0.07)

EAPC, estimated annual percentage change; SDI, sociodemographic index; UI, uncertainty interval.

#### Global DALYs

The global number of OM-associated DALYs in children increased by 6.84% from 1990–2021 (969,453; 95% UI, 564,403–1,487,926) in 1990 vs. 1,035,749 (95% UI, 590,162–1,610,005) in 2021; but the EAPC was −0.19 (95% CI, −0.23 to −0.15) (Supplementary eTable S1 in Supplementary Material). From 1990–2021, the number of DALYs related to OM in children of all age groups increased, but it showed a downward trend in children under 2 years old (<1 year: −38.57%; 12–23 months: −7.73%). The greatest increase in the number of OM associated DALYs (16.64%) was observed among children aged 10–14 years. The groups with the highest numbers of OM associated DALYs in both 1990 and 2021 were children aged 5–9 years (340,810 and 386,365, respectively). In 2021, the rate of OM associated DALYs among children was higher in boys than in girls (0–14 years: boys, 54.34; girls, 48.43). Children under 1 year old had the lowest rate of OM associated DALYs (boys: 35.87; Girls: 33.45) (Figure 1B).

### OM in children: SDI regional trends

#### Incidence trends by SDI level

**Figure 2:**
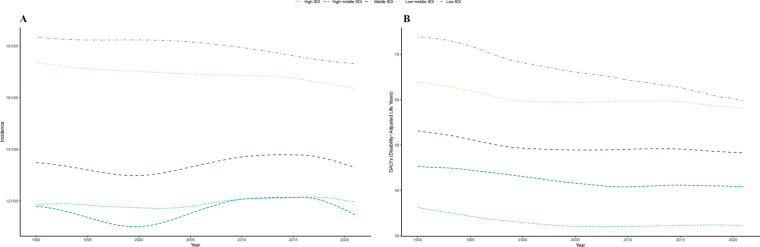
Epidemiologic trends of incidence and disability-adjusted life-years (DALYs) rates in 5 sociodemographic index (SDI) regions of childhood otitis media from 1990 to 2021. **(A)** Trends in incidence rate. **(B)** Trends in DALYs rate.

The low-middle SDI region had the most cases of childhood OM in 2021 (94,821,611; 95% UI, 65,156,518–138,188,242). The incident cases in the low SDI region increased by 89.71% (95% UI, 85.88%–93.75%). The greatest increase in the incidence of childhood OM occurred in the high SDI region (EAPC, 0.20; 95% CI, 0.09–0.32) (Table 1 and Figure 2A).

#### DALYs across SDI regions

DALYs In 2021, the low-middle SDI region had the highest number of OM associated DALYs (336,882; 95% UI, 192,178–522,382) with a increase of 11.64% from 1990–2021. The high middle SDI region had the greatest decrease (24.07%) in the number of OM associated DALYs (Supplementary eFigure S2B and Supplementary Table S1 in the Supplementary Material).

### OM in children: geographic regional trends

#### Incidence variation by WHO regions

**Figure 3:**
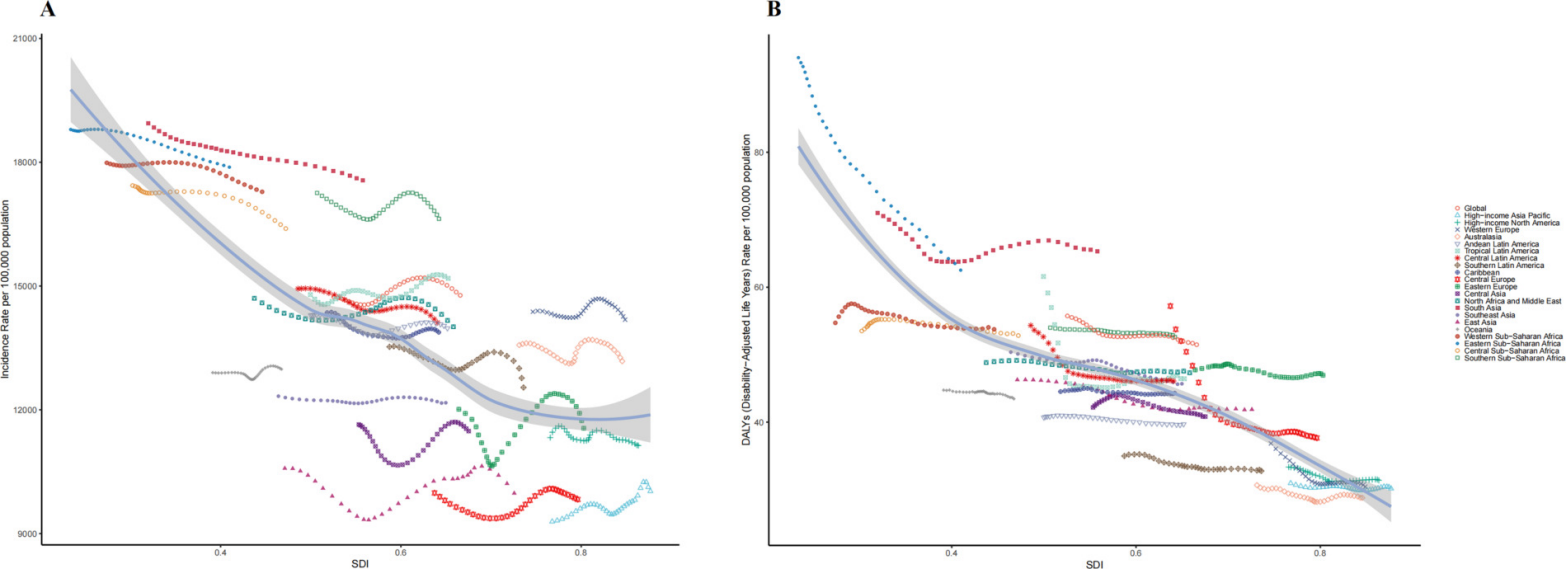
Incidence and disability-adjusted life-years (DALYs) rates for childhood otitis media from 1990 to 2021. **(A)** Incidence rate. **(B)** DALYs rate.

Incidence Among 21 geographic regions, South Asia had the most cases of childhood OM in 2021 (89,044,430; 95% UI, 60,926,931–130,405,120), whereas Oceania had the fewest (659,744; 95% UI, 450,075–967,314). The incidence of childhood OM was highest in Eastern Sub-Saharan Africa (17,880.67; 95% UI, 12,140.06–25,784.82), mainly in children between 12 and 23 months of age, and the incidence of OM in boys is higher than that in girls. In contrast, the incidence of childhood OM was lowest in Central Europe (9,827.73; 95% UI, 6,932.53–13,755.35). From 1990–2021, Eastern Europe had the largest increase in the incidence of childhood OM (EAPC, 0.29; 95% CI, 0.12–0.47), whereas South Asia had the largest decrease (EAPC, −0.21; 95% CI, −0.22–0.20) (Table 1). In 2021, Eastern Sub-Saharan Africa (SDI, 0.41) had the highest incidence of childhood OM, whereas Central Europe (SDI, 0.80) had the lowest incidence. The global SDI was 0.67 in 2021; 6 regions (e.g., Eastern Sub-Saharan Africa and South Asia) had higher incidences of childhood OM than the global mean, whereas 15 regions (e.g., Central Europe and East Asia) had lower incidences than the global mean (14,774.55) (Figure 3A).

#### Regional DALYs distribution

DALYs In 2021, South Asia had the highest number of childhood OM associated DALYs (331,268; 95% UI, 189,150–509,463), whereas Australasia had the lowest number (1,650; 95% UI, 886–2,694). South Asia had the highest DALYs rate (65.34; 95% UI, 37.31–100.48); Australasia had the lowest DALYs rate (28.78; 95% UI, 15.46–47.01). From 1990–2021, High-income Asia Pacific had the smallest decrease in the DALYs rate (EAPC, −0.047; 95% CI, −0.08 to −0.02); Eastern Sub-Saharan Africa had the largest decrease (EAPC, −1.33; 95% CI, −1.35 to −1.31). 5 regions (e.g., South Asia and Eastern Sub-Saharan Africa) had rates of DALYs that were higher than the global mean, whereas 16 regions (e.g., Australasia and High-income Asia Pacific) had rates that were lower than the global mean (51.48) (Figure 3B and Supplementary eTable S1 in Supplementary Material).

### OM in children: national trends

#### Incidence

**Figure 4:**
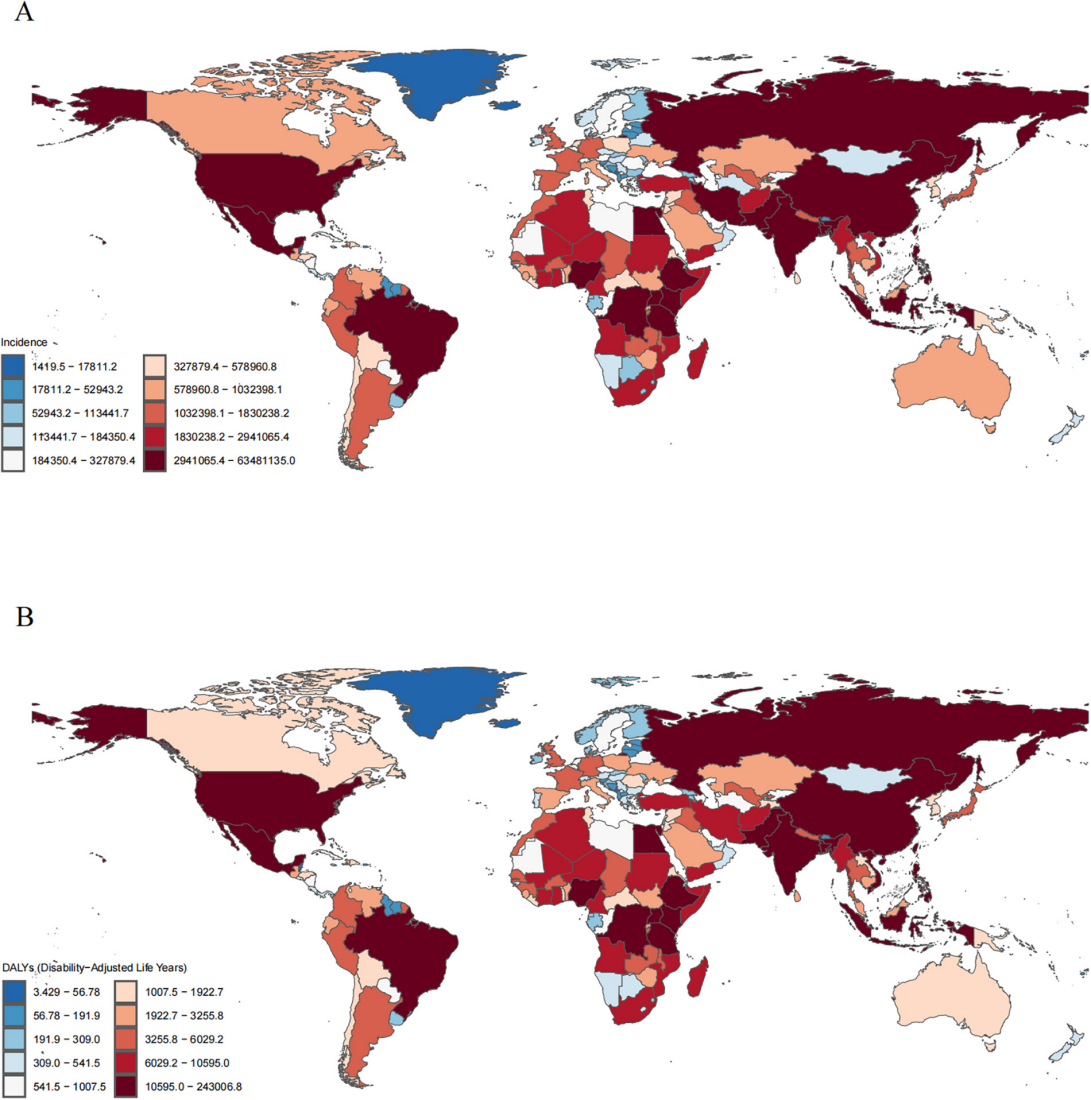
Incident and disability-adjusted life-years (DALYs) cases of otitis media in children in 204 countries and territories. **(A)** Incidence cases. **(B)** DALYs cases.

In 2021, among 204 countries, India had the most cases of childhood OM (63,481,135; 95% UI, 43,307,190–92,849,439); Pakistan had the highest incidence rate of childhood OM (19,045.75; 95% UI, 12,952.13–27,589.47) (Figure 4A and Supplementary eFigure S1A in the Supplementary Material). Republic of Korea (EAPC, 0.43; 95% CI, 0.14–0.73) had the largest increases in childhood OM incidence; Northern Mariana Islands (EAPC, −0.70; 95% CI, −0.84 to −0.57) had the largest decreases (Supplementary eFigure S2A in the Supplementary Material). In 2021, Pakistan (SDI, 0.50) had the highest incidence of childhood OM, whereas Taiwan (Province of China) (SDI, 0.87) had the lowest incidence. The global incidence of childhood OM in 2021 was 14,774.55 (95% UI, 21,459.04- 10,199.43); the incidences were above the global mean in 58 countries and below the global mean in 146 countries (Supplementary eFigure S3A in the Supplementary Material).

#### DALYS

In 2021, India had the highest number of OM-associated childhood DALYs (243,007; 95% UI, 373,596–138,945). Somalia had the highest rate of childhood OM associated DALYs (88.49; 95% UI, 42.07–199.81) (Figure 4B and Supplementary eFigure S1B in the Supplementary Material). State of Libya (EAPC, 0.17; 95% CI, 0.13–0.22) had the greatest increase in DALYs rate; Hungary (EAPC, −1.98; 95% CI, −2.60 to −1.35) had the greatest decreases (Supplementary eFigure S2B in the Supplementary Material). Somalia (SDI, 0.08) had the highest rate of childhood OM associated DALYs; Monaco (SDI, 0.91) had the lowest rate. The global rate of childhood OM associated DALYs in 2021 was 51.48 (95% UI, 29.33–80.03); the rates were above the global mean in 42 countries and below the global mean in 162 countries (Supplementary eFigure S3B in the Supplementary Material).

#### Risk factors for OM in children

Secondhand smoke, particulate matter pollution, low birth weight, and short gestation were identified as significant contributors to the global DALYs of OM in children. Among these, secondhand smoke was the leading risk factor, accounting for 7.66% of DALYs (Supplementary eFigure S4 in the Supplementary Material). Identification and effective management of these key risk factors could facilitate the development of targeted prevention strategies to reduce the burden of OM.

#### Predictions of OM from 2020 to 2035

**Figure 5:**
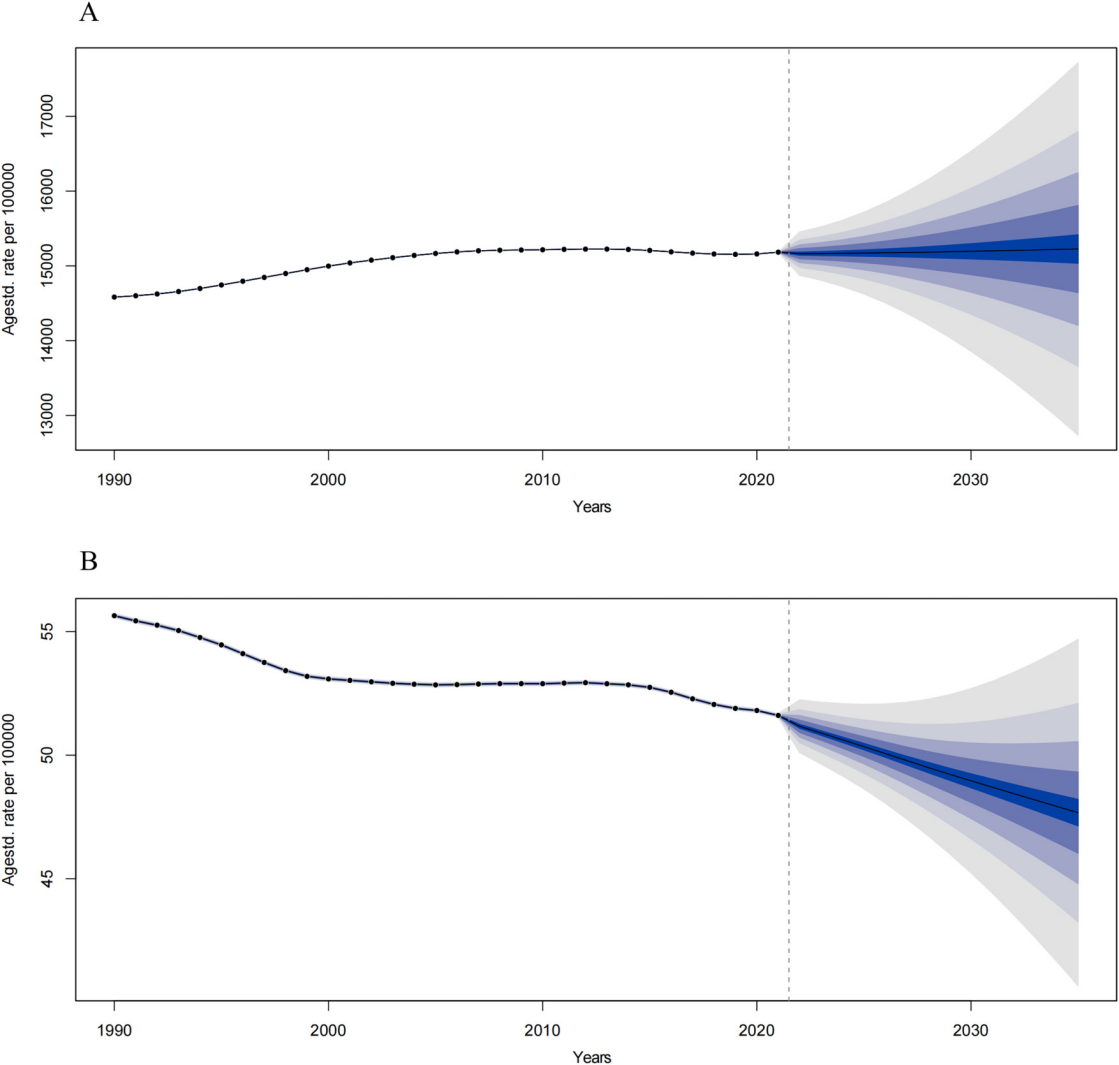
Predict the incidence and DALYs of otitis media in global in 2035. **(A)** The trends of incidence from 2022 to 2035. **(B)** The trends of DALYs from 2022 to 2035. The blue region shows the upper and lower limits of the 95% uncertainty intervals (95% UIs).

Using the Bayesian Age-Period-Cohort (BAPC) model, we projected global age-standardized incidence rates (ASIR) and DALYs for childhood OM from 2020–2035. By 2035, the predicted global age-standardized incidence rate is expected to reach 15,225.78 per 100,000 population, representing a 0.28% increase from 2021 levels (Figure 5A). Additionally, the predicted global age-standardized DALY rate in 2035 is estimated to be 42.67 per 100,000 population, reflecting a 7.63% increase from 2021 (Figure 5B).

## Discussion

This study offers a comprehensive analysis of the global, regional, and national burden of childhood OM by utilizing data from the GBD database for the period 1990–2021. The findings indicate an increase in the ASIR of childhood OM, alongside a decrease in the global age-standardized DALY rate over recent decades. However, substantial regional and national disparities persist. These results are largely consistent with previous studies (17), but by extending the data to 2021, this study presents the most up-to-date epidemiological trends and risk factor analyses, addressing the gap in data from 2019–2021. This research provides valuable insights into the burden of childhood OM across regions with varying income levels, supporting policymakers and clinicians in formulating effective prevention and management strategies.

From 1990–2021, DALYs related to OM in children decreased globally, despite a 15.97% increase in the incidence of OM during this period. The incidence of OM has risen across all pediatric age groups, with this trend being particularly marked in low- and lower-middle-income regions. These areas are often characterized by insufficient medical resources, limited public health service coverage, and low socioeconomic development. High-incidence countries frequently face challenges such as scarce healthcare resources, inadequate health service coverage, and prevalent high-risk behaviors (e.g., exposure to secondhand smoke, air pollution). Previous studies have highlighted racial, ethnic, and socioeconomic disparities in the prevalence and management of OM (18–20). Our findings further identify regions and countries with a higher burden of OM and corresponding deficiencies in healthcare resources. In regions with lower economic development, improving access to healthcare services is critical, including expanding the number of medical facilities, particularly in rural or underserved areas. Recommendations for targeted interventions and rehabilitation should be tailored based on local resources, infrastructure, cultural context, and socioeconomic conditions. Therefore, a comprehensive evaluation of the local healthcare system—facilitating collaboration between policymakers, healthcare professionals, and other stakeholders—is essential to develop and implement effective strategies.

Furthermore, we identified a significant inverse association between the SDI and both the incidence of OM and the number of associated DALYs. The most substantial reduction in DALYs related to OM in children was observed in regions with High-middle SDI, followed by those with high SDI. This decline is likely due to better access to healthcare services in these areas, enabling earlier diagnosis and more effective treatment of OM in children. In contrast, the increase in DALYs in low SDI regions highlights significant deficiencies in the prevention and management of OM, likely attributable to limited healthcare resources, poor access to services, and challenging socioeconomic conditions. Lower socioeconomic status and reliance on public insurance have been associated with missed otolaryngology appointments and worse hearing outcomes (21, 22). Additionally, children from socioeconomically disadvantaged backgrounds are less likely to receive antibiotic treatment for OM and more likely to experience complications (10, 11). Future initiatives should prioritize fostering regional collaboration to integrate medical advancements from high-income countries into low- and middle-income countries (LMICs), thereby enhancing healthcare capacity. Furthermore, addressing disparities in pediatric otolaryngology should extend beyond race and socioeconomic status to include factors such as insurance coverage, caregiver education, primary language, and social support. These efforts are crucial for reducing the global burden of childhood OM (23).

This study confirms that secondhand smoke, particulate matter pollution, low birth weight, and short gestation are significant risk factors for OM. Understanding the biological mechanisms through which these factors operate may provide important insights for public health interventions. Secondhand smoke (SHS) contains a complex mixture of harmful chemicals that can impair the mucociliary function of the Eustachian tube and increase nasopharyngeal colonization by pathogenic bacteria. SHS exposure is associated with local inflammation, increased mucus production, and structural changes in the respiratory epithelium, which predispose children to recurrent OM episodes. Nicotine and other toxins in SHS have also been shown to modulate the host immune response, reducing the effectiveness of innate immunity in clearing infections (24–26). Ambient particulate matter (PM2.5 and PM10) has been implicated in respiratory tract inflammation and epithelial barrier dysfunction. PM exposure can activate Toll-like receptors (TLRs) and the NLRP3 inflammasome pathway, leading to upregulation of pro-inflammatory cytokines (IL-6, IL-8, TNF-α), which may extend to the middle ear via the Eustachian tube. Additionally, particles may act as carriers for microbial components, facilitating bacterial adhesion and biofilm formation in the middle ear cavity (27, 28). Low birth weight (LBW) infants often exhibit delayed maturation of immune function, particularly involving neutrophil activity and secretory IgA production. This immunologic immaturity compromises mucosal defense mechanisms and increases susceptibility to upper respiratory tract infections, a key precursor for OM (29). LBW is also associated with anatomical immaturity of the Eustachian tube, increasing the likelihood of its dysfunction. Preterm birth has been independently associated with a higher risk of OM, likely due to underdeveloped structural and immunologic systems. Premature infants often have shorter and more horizontal Eustachian tubes, less efficient drainage, and reduced ciliary function. Moreover, their immature immune responses-particularly lower levels of maternal IgG transfer-make them more vulnerable to early and recurrent infections (30, 31). These findings suggest that the identified risk factors contribute to OM via both direct inflammatory pathways and indirect immunologic or anatomical vulnerabilities. Public health policies aimed at reducing exposure to environmental pollutants and improving maternal and neonatal care may play a critical role in mitigating the global burden of pediatric OM.

### Model assumptions and potential bias

Although GBD research is extensive and comprehensive, certain limitations concerning data quality and integrity warrant acknowledgment. The reliability of the estimates can be influenced by disparities in data collection methodologies and the uneven availability of high-quality data across countries. In low SDI regions, data sources are frequently constrained or outdated, resulting in a heightened reliance on simulation-based estimates. This may introduce uncertainty or bias, potentially compromising the accuracy and comparability of the findings. Additionally, potential biases arising from missing data, underreporting, or diagnostic variability could affect the interpretation of trends. While GBD employs a standardized methodology and provides a 95% uncertainty interval to account for these discrepancies, the generalizability of the results should be interpreted with caution, particularly in areas with inadequate monitoring infrastructure. Future initiatives should prioritize enhancing primary data collection and reporting systems in resource-limited settings to improve the robustness and effectiveness of global disease burden estimation.

The Bayesian age-period-cohort (BAPC) model used in this study assumes a stable data structure across time and regions. To minimize overfitting or imprecision, we ensured that each age-period stratum had sufficient sample size (*n* ≥ 5). Convergence diagnostics indicated that all chains reached stable posterior distributions (R-hat < 1.05; ESS > 1,000), supporting the reliability of model estimates. Sensitivity analyses using varying priors further confirmed the robustness of the projected incidence and DALYs trends. Despite these efforts, some limitations remain. First, the model does not account for all unmeasured confounding factors that may influence both exposure (e.g., secondhand smoke, PM2.5) and outcomes (OM burden). For example, household hygiene conditions, parental health literacy, and access to primary care may significantly differ across regions and affect both the exposure risk and early diagnosis of OM. These factors are not directly captured in the GBD dataset, which may introduce residual bias. Additionally, inequities in healthcare infrastructure, particularly in low- and middle-income countries, may result in underreporting of cases or misclassification of disease burden. Variations in health information systems, otologic care access, vaccination coverage, and antibiotic use further complicate global comparisons. Future research incorporating subnational healthcare data or survey-based validation studies may help address these gaps and refine burden estimation and forecasting.

## Conclusions

In conclusion, OM in children represents a significant global public health challenge. From 1990–2021, the incidence of OM in children has continued to rise globally. Despite a downward trend in the DALY rate for OM among children over this period, regional disparities remain evident. Notably, both the incidence and DALY rates of OM in children are still high in low SDI regions. These findings may assist policymakers in justifying the allocation of medical resources, particularly in low SDI countries. There is an urgent need for healthcare professionals to develop more cost-effective and targeted strategies to reduce the morbidity associated with childhood OM, alleviate the socioeconomic burden, and mitigate the associated risks.

## Data Availability

The original contributions presented in the study are included in the article/Supplementary Material, further inquiries can be directed to the corresponding authors.
