# Supplementary material for: Global, regional, and national epidemiology of otitis media in children from 1990 to 2021
Source: Front Pediatr. 2025 Jul 2;13:1513629. doi: 10.3389/fped.2025.1513629 (PMC12263564; doi:10.3389/fped.2025.1513629)
Supplement: Supplementary file 1 [file Datasheet1.docx]

**Supplementary Online Content**

**eFigure 1.** The incidence and DALYs rates of otitis media in children in 204 countries and territories.

**eFigure 2.** The national burden of otitis media in children in 204 countries and territories.

**eFigure 3.** Incidence and DALYs rates of otitis media in children in 204 countries by SDI in 2021.

**eFigure 4.** Percentage of DALYs due to otitis media attributable to each risk factor for the Global Burden of Disease regions.

**eTable 1.** DALYs of otitis media in children between 1990 and 2021 at the global and regional levels.

This supplementary material has been provided by the authors to give readers additional information about their work.


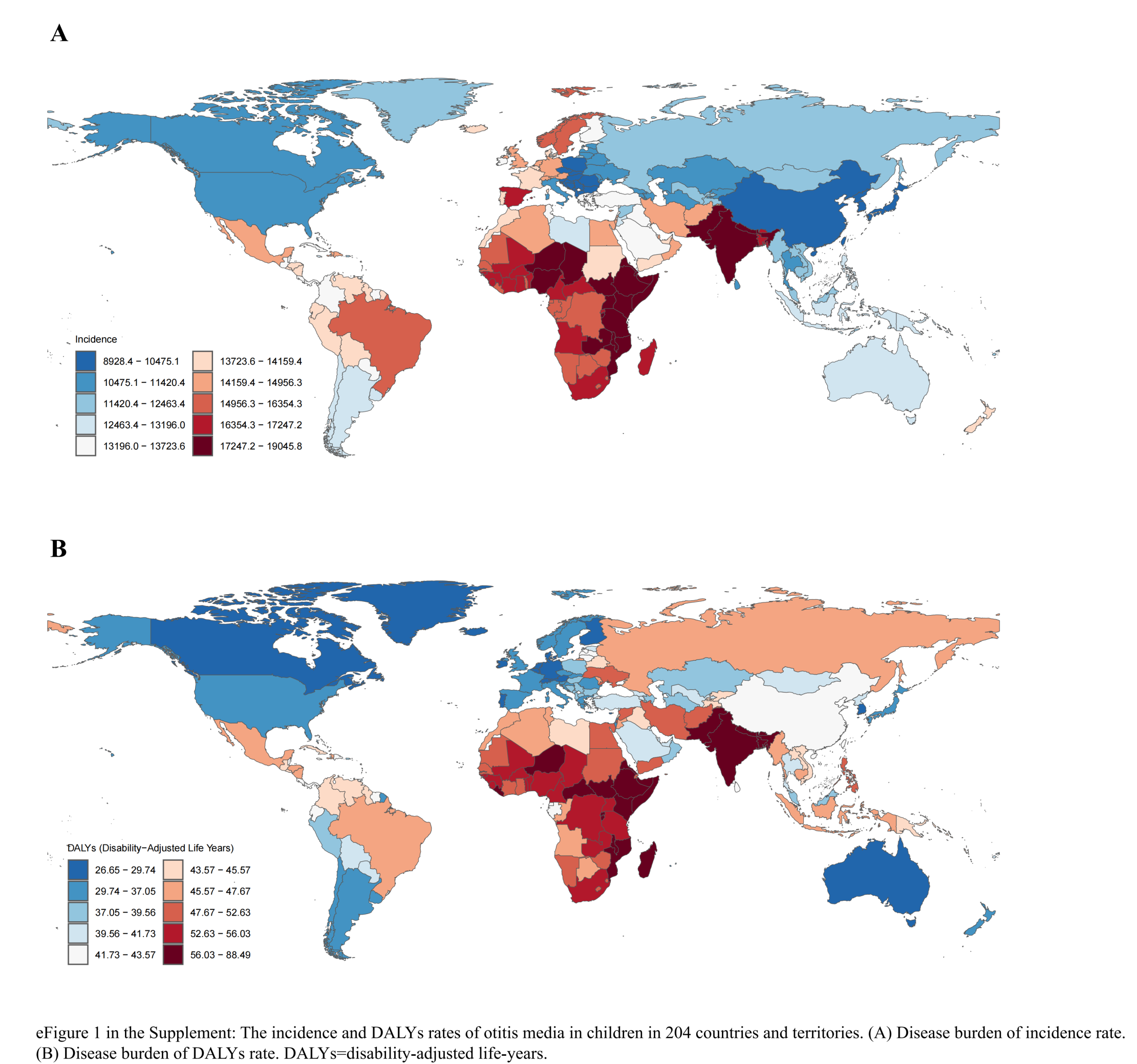


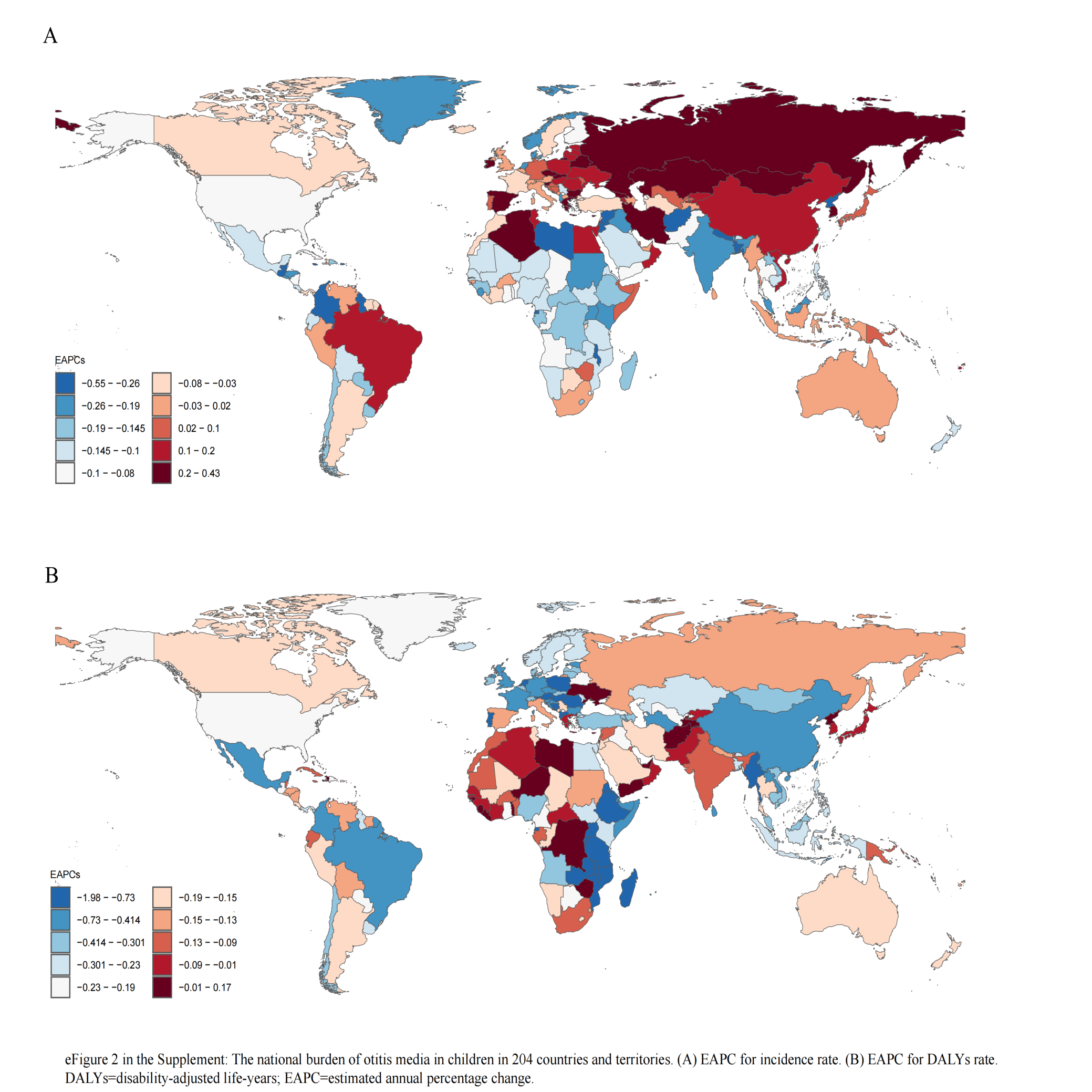


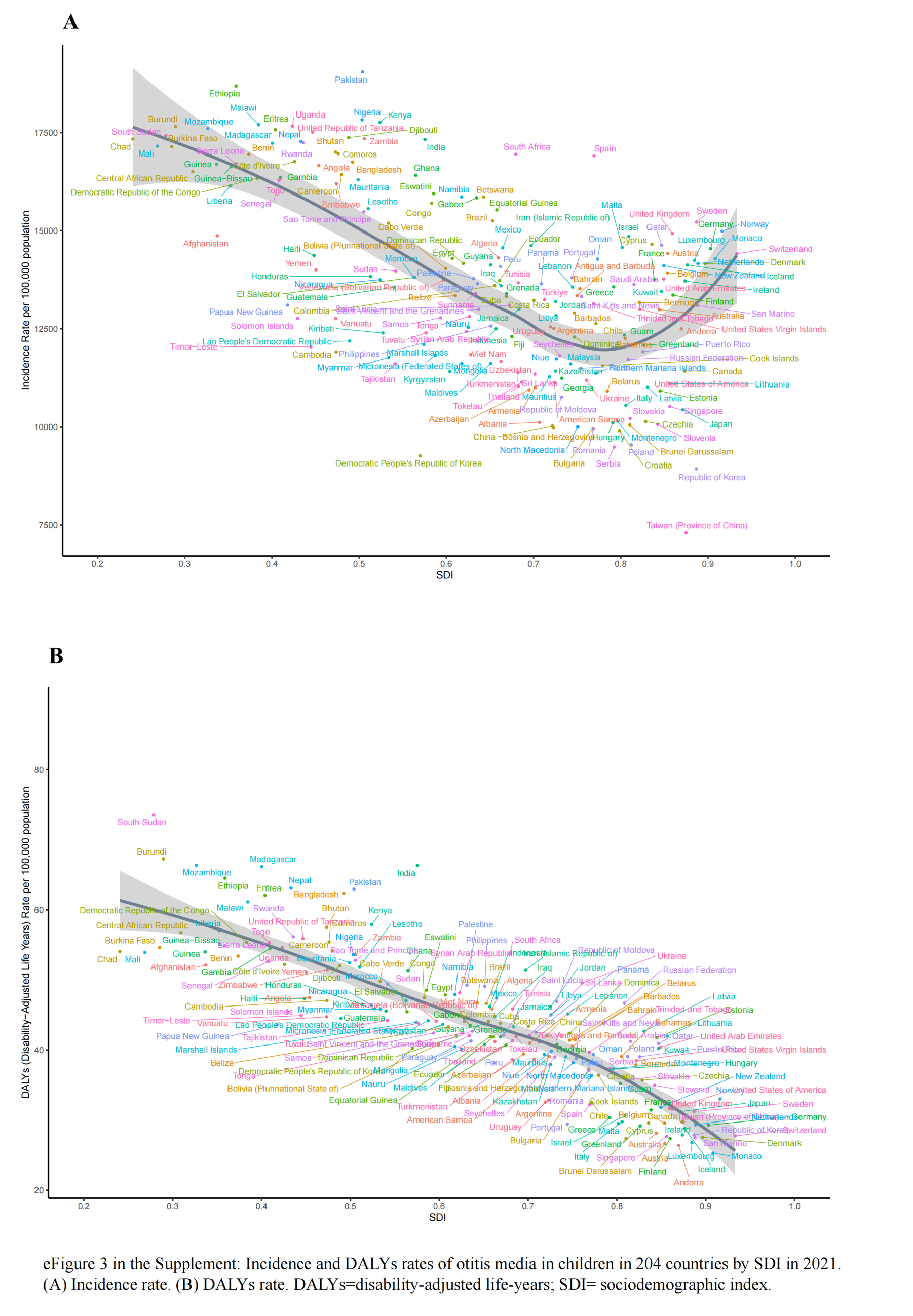


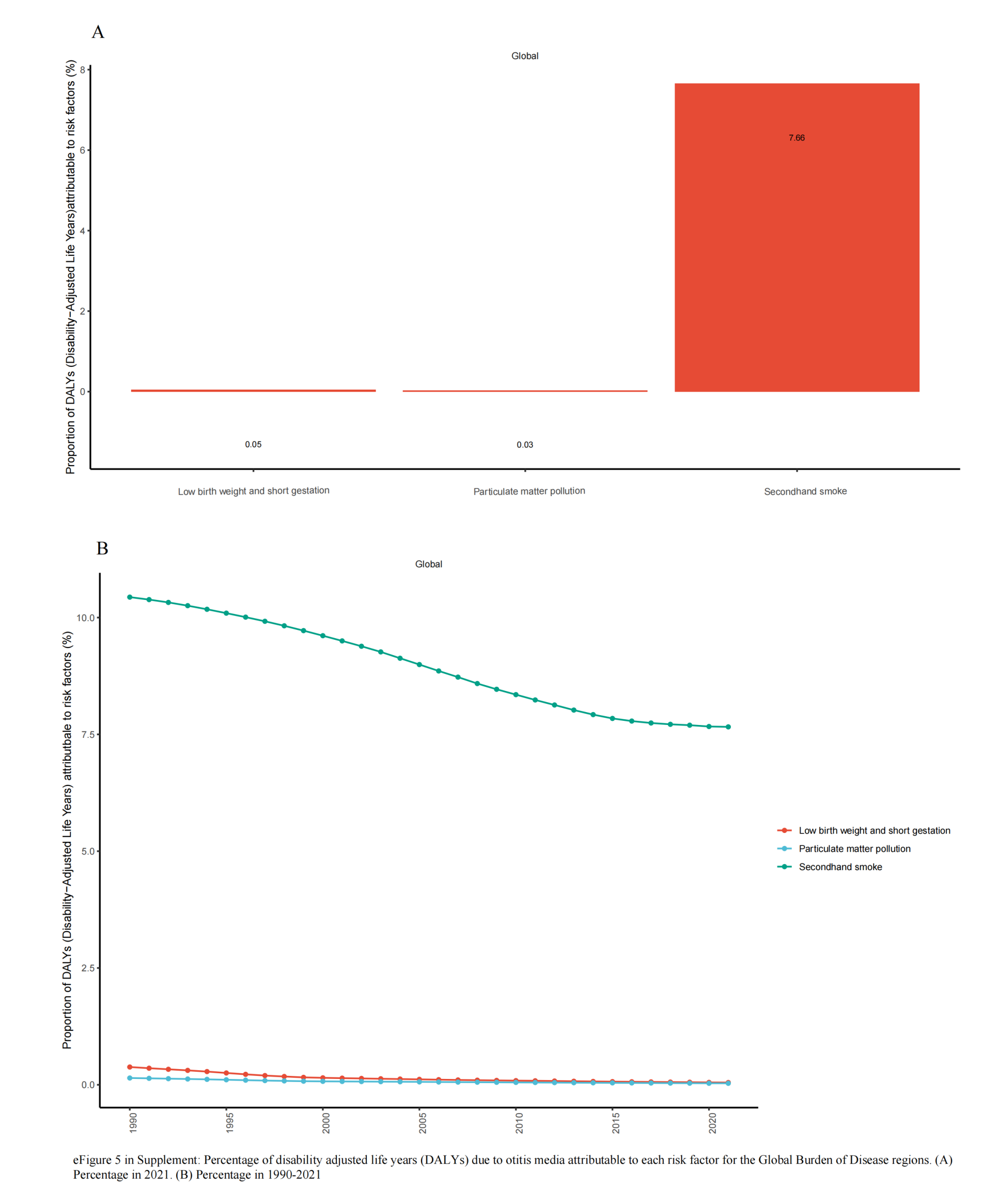


eTable 1 in the Supplement. DALYs of otitis media in children between 1990 and 2021 at the global and regional levels.

| location | Rate per 100,000 (95% UI) | | | | | |
| --- | --- | --- | --- | --- | --- | --- |
|  | 1990 | | 2021 | | 1990-2021 | |
|  | DALYs cases | DALYs rate | DALYs cases | DALYs rate | Cases change | EAPC |
| Global | 969452.98  (564402.87 to 1487925.82) | 55.74  (32.45 to 85.55) | 1035749.04  (590161.89 to 1610004.58) | 51.48  (29.33 to 80.03) | 0.07  (0.00 to 0.11) | -0.19  (-0.23 to -0.15) |
| High SDI | 67331.14  (39893.89 to 104342.07) | 36.24  (21.47 to 56.16) | 55451.13  (30878.04 to 89081.89) | 32.14  (17.90 to 51.63) | -0.18  (-0.14 to -0.23) | -0.32  (-0.40 to -0.23) |
| High-middle SDI | 124025.94  (73340.50 to 193064.53) | 45.33  (26.80 to 70.56) | 94177.27  (53856.71 to 149594.63) | 40.79  (23.33 to 64.79) | -0.24  (-0.22 to -0.27) | -0.37  (-0.43 to -0.31) |
| Middle SDI | 306551.87  (181607.35 to 473176.10) | 53.11  (31.46 to 81.98) | 273852.06  (156407.87 to 431723.46) | 48.31  (27.59 to 76.16) | -0.11  (-0.08 to -0.15) | -0.24  (-0.30 to -0.18) |
| Low-middle SDI | 301748.96  (173499.43 to 461575.45) | 63.91  (36.75 to 97.77) | 336882.22  (192178.69 to 522382.92) | 58.10  (33.14 to 90.09) | 0.12  (0.05 to 0.16) | -0.23  (-0.28 to -0.18) |
| Low SDI | 169030.13  (97085.58 to 250483.33) | 73.84  (42.41 to 109.42) | 274709.48  (154120.04 to 431570.28) | 59.69  (33.49 to 93.77) | 0.63  (0.29 to 0.85) | -0.68  (-0.70 to -0.65) |
| Andean Latin America | 6043.98  (3350.00 to 9687.29) | 40.69  (22.56 to 65.23) | 7183.20  (4056.92 to 11428.51) | 39.70  (22.42 to 63.16) | 0.19  (0.13 to 0.24) | -0.13  (-0.14 to -0.11) |
| Australasia | 1406.13  (786.60 to 2245.16) | 30.66  (17.15 to 48.96) | 1649.49  (885.76 to 2694.46) | 28.78  (15.46 to 47.01) | 0.17  (0.08 to 0.26) | -0.16  (-0.23 to -0.09) |
| Caribbean | 5081.53  (2881.33 to 8005.45) | 44.53  (25.25 to 70.15) | 5095.82  (2874.29 to 8095.44) | 44.29  (24.98 to 70.36) | 0.00  (-0.03 to 0.04) | -0.05  (-0.07 to -0.04) |
| Central Asia | 10542.70  (5954.88 to 16643.78) | 42.19  (23.83 to 66.60) | 11297.96  (6415.15 to 17935.56) | 40.82  (23.18 to 64.81) | 0.07  (0.03 to 0.11) | -0.21  (-0.27 to -0.15) |
| Central Europe | 16866.63  (11501.04 to 24325.63) | 57.21  (39.01 to 82.51) | 6664.73  (3856.32 to 10718.02) | 37.65  (21.79 to 60.55) | -0.06  (-0.55 to -0.68) | -0.99  (-1.26 to -0.73) |
| Central Latin America | 34982.17  (21754.67 to 52649.12) | 54.34  (33.79 to 81.78) | 29259.92  (16773.85 to 46329.17) | 46.09  (26.42 to 72.98) | -0.16(-0.25 to -0.11) | -0.45  (-0.57 to -0.33) |
| Central Sub-Saharan Africa | 13538.38  (7737.29 to 21548.34) | 53.51  (30.58 to 85.18) | 31003.61  (17872.28 to 49240.69) | 52.83  (30.46 to 83.91) | 1.29  (1.09 to 1.46) | -0.08  (-0.13 to -0.04) |
| East Asia | 152650.63  (88306.85 to 240734.87) | 46.28  (26.77 to 72.99) | 111963.96  (64846.80 to 176785.33) | 41.88  (24.26 to 66.12) | -0.27  (-0.30 to -0.24) | -0.40  (-0.46 to -0.34) |
| Eastern Europe | 24567.78  (14275.51 to 38104.89) | 47.74  (27.74 to 74.04) | 16643.59  (9761.72 to 26085.90) | 46.96  (27.54 to 73.60) | -0.32  (-0.34 to -0.31) | -0.13  (-0.16 to -0.09) |
| Eastern Sub-Saharan Africa | 85163.52  (46260.22 to 138264.98) | 94.03  (51.08 to 152.66) | 111568.41  (60322.86 to 171870.76) | 62.53  (33.81 to 96.32) | 0.31  (-0.08 to 0.70) | -1.33  (-1.35 to -1.31) |
| High-income Asia Pacific | 10891.61  (6167.32 to 17320.81) | 30.94  (17.52 to 49.21) | 6762.64  (3758.93 to 10822.44) | 30.16  (16.76 to 48.26) | -0.38  (-0.40 to -0.34) | -0.05  (-0.08 to -0.02) |
| High-income North America | 20565.32  (12120.64 to 32337.99) | 33.34  (19.65 to 52.43) | 20594.96  (11826.90 to 33173.82) | 31.39  (18.02 to 50.56) | 0.00  (-0.05 to 0.04) | -0.21  (-0.26 to -0.16) |
| North Africa and Middle East | 68614.71  (39033.98 to 107992.43) | 48.84  (27.79 to 76.87) | 86789.51  (49111.93 to 136640.72) | 47.34  (26.79 to 74.54) | 0.26  (0.23 to 0.29) | -0.14  (-0.16 to -0.12) |
| Oceania | 1199.13  (689.84 to 1884.10) | 44.75  (25.74 to 70.31) | 2208.13  (1250.98 to 3439.79) | 43.46  (24.62 to 67.70) | 0.84  (0.73 to 0.96) | -0.07  (-0.08 to -0.05) |
| South Asia | 307647.95  (177621.50 to 468046.14) | 70.99  (40.99 to 108.00) | 331267.86  (189149.52 to 509463.28) | 65.34  (37.31 to 100.48) | 0.08  (0.00 to 0.13) | -0.13  (-0.24 to -0.01) |
| Southeast Asia | 86021.74  (49058.50 to 134517.20) | 50.38  (28.73 to 78.78) | 78886.84  (44640.39 to 125028.16) | 45.69  (25.86 to 72.42) | -0.08  (-0.12 to -0.05) | -0.29  (-0.33 to -0.26) |
| Southern Latin America | 5221.71  (2895.08 to 8303.48) | 34.98  (19.40 to 55.63) | 4749.50  (2637.26 to 7569.84) | 32.77  (18.19 to 52.22) | -0.09  (-0.15 to -0.04) | -0.24  (-0.28 to -0.20) |
| Southern Sub-Saharan Africa | 11173.23  (6444.02 to 17478.37) | 54.00  (31.15 to 84.48) | 12661.71  (7214.43 to 19905.12) | 52.61  (29.98 to 82.71) | 0.13  (0.09 to 0.17) | -0.06  (-0.07 to -0.06) |
| Tropical Latin America | 33022.00  (21830.94 to 47653.51) | 61.59  (40.72 to 88.88) | 23305.51  (13535.19 to 36391.23) | 46.43  (26.97 to 72.50) | -0.29  (-0.39 to -0.22) | -0.50  (-0.77 to -0.23) |
| Western Europe | 26165.81  (15765.94 to 39914.13) | 36.84  (22.20 to 56.20) | 20728.58  (11331.97 to 33294.99) | 30.43  (16.64 to 48.88) | -0.21  (-0.29 to -0.16) | -0.45  (-0.57 to -0.33) |
| Western Sub-Saharan Africa | 48086.32  (27827.89 to 75149.25) | 54.72  (31.67 to 85.51) | 115463.12  (66732.89 to 181197.06) | 53.76  (31.07 to 84.37) | 1.40  (1.35 to 1.45) | -0.21  (-0.26 to -0.16) |

Abbreviations: EAPC, estimated annual percentage change; SDI, Sociodemographic Index; UI, uncertainty interval.
